# Supplementary figures and images for: Effect of prolactin on cytotoxicity and oxidative stress in ovine ovarian granulosa cells
Source: PeerJ. 2023 Jul 10;11:e15629. doi: 10.7717/peerj.15629 (PMC10340108; doi:10.7717/peerj.15629)

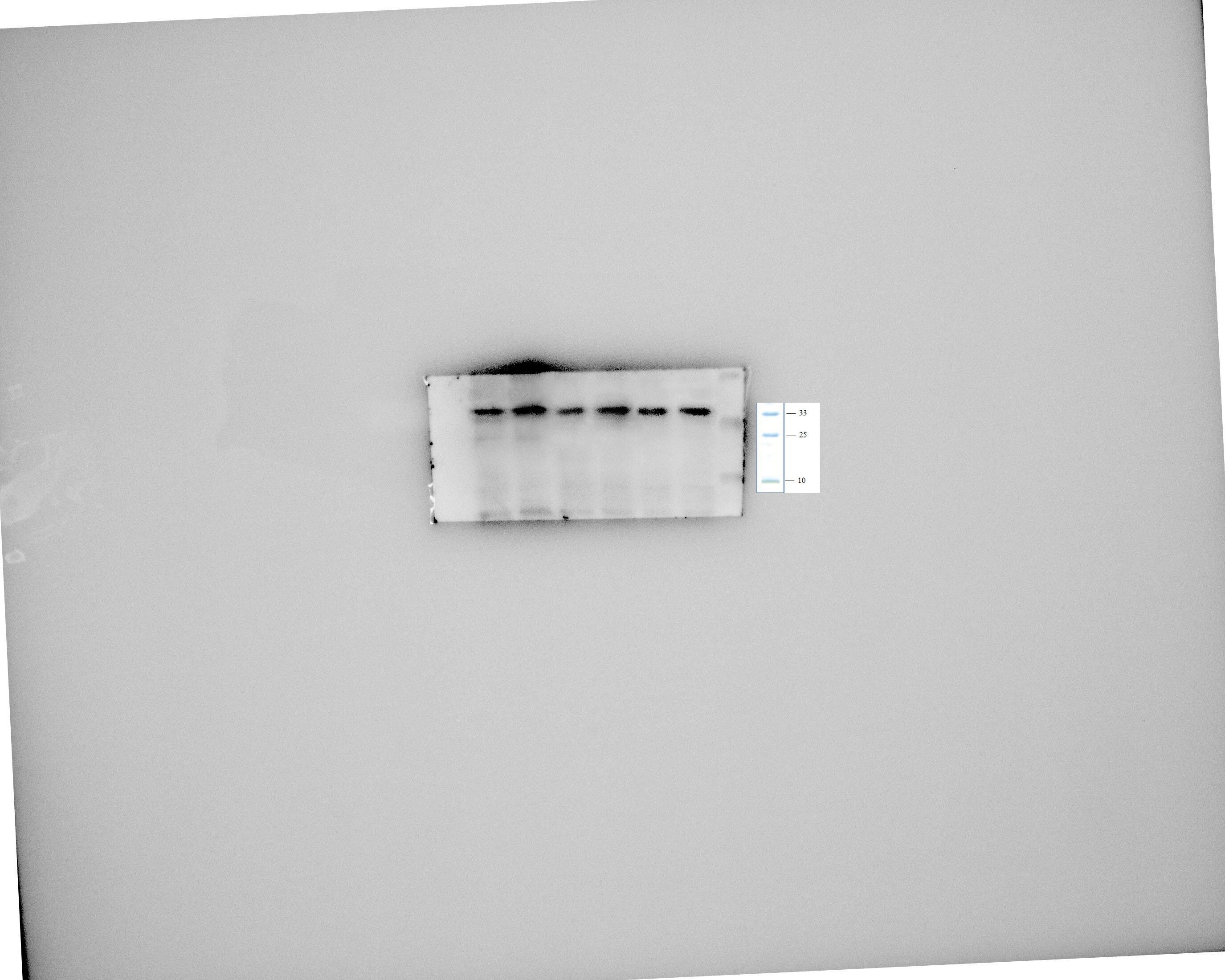

Supplement: Supplemental Information 5 [file peerj-11-15629-s005.zip › WB -Uncropped/BCL2L1 ┐╜▒┤.jpg]

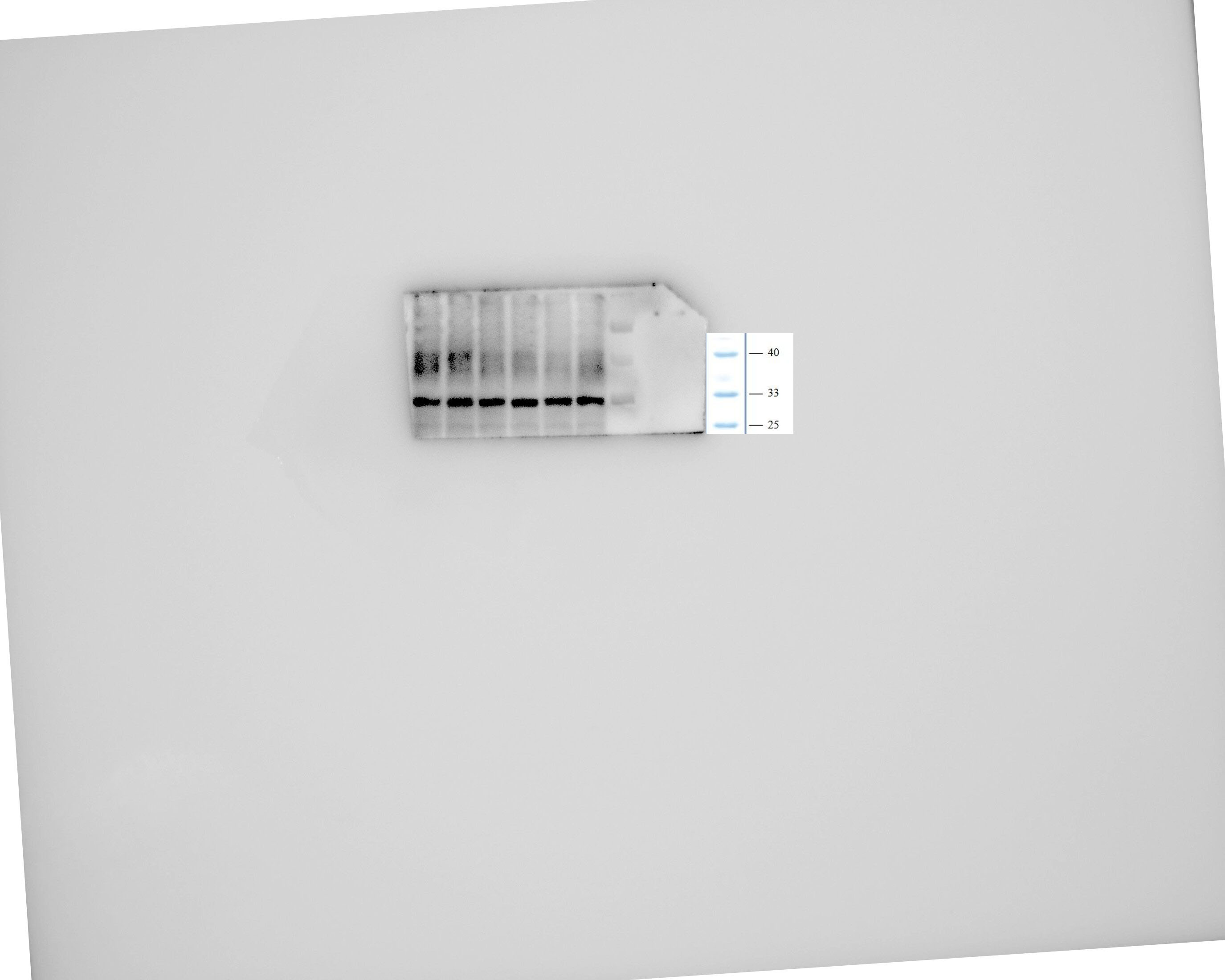

Supplement: Supplemental Information 5 [file peerj-11-15629-s005.zip › WB -Uncropped/GAPDH1 ┐╜▒┤.jpg]

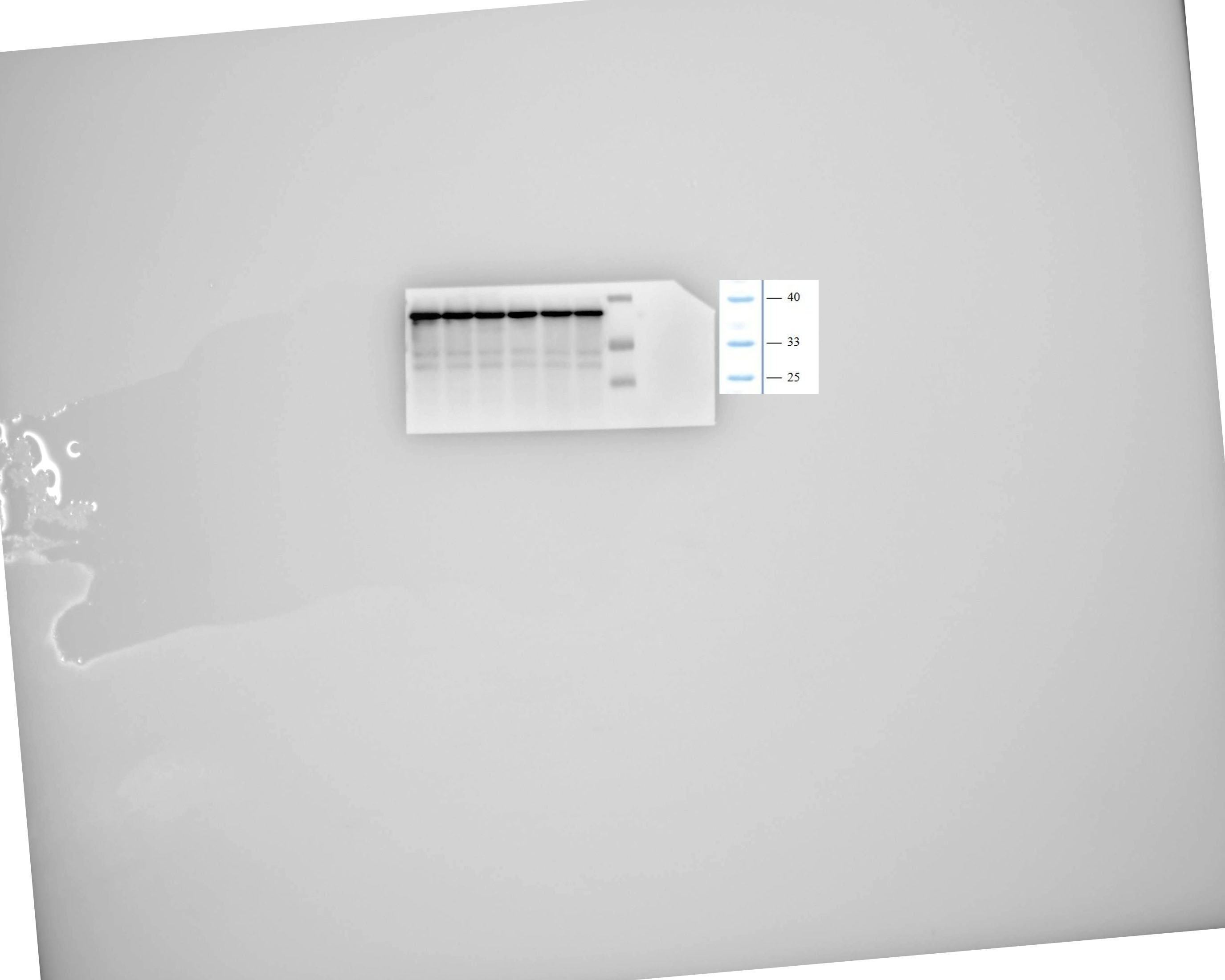

Supplement: Supplemental Information 5 [file peerj-11-15629-s005.zip › WB -Uncropped/GAPDH2 ┐╜▒┤.jpg]

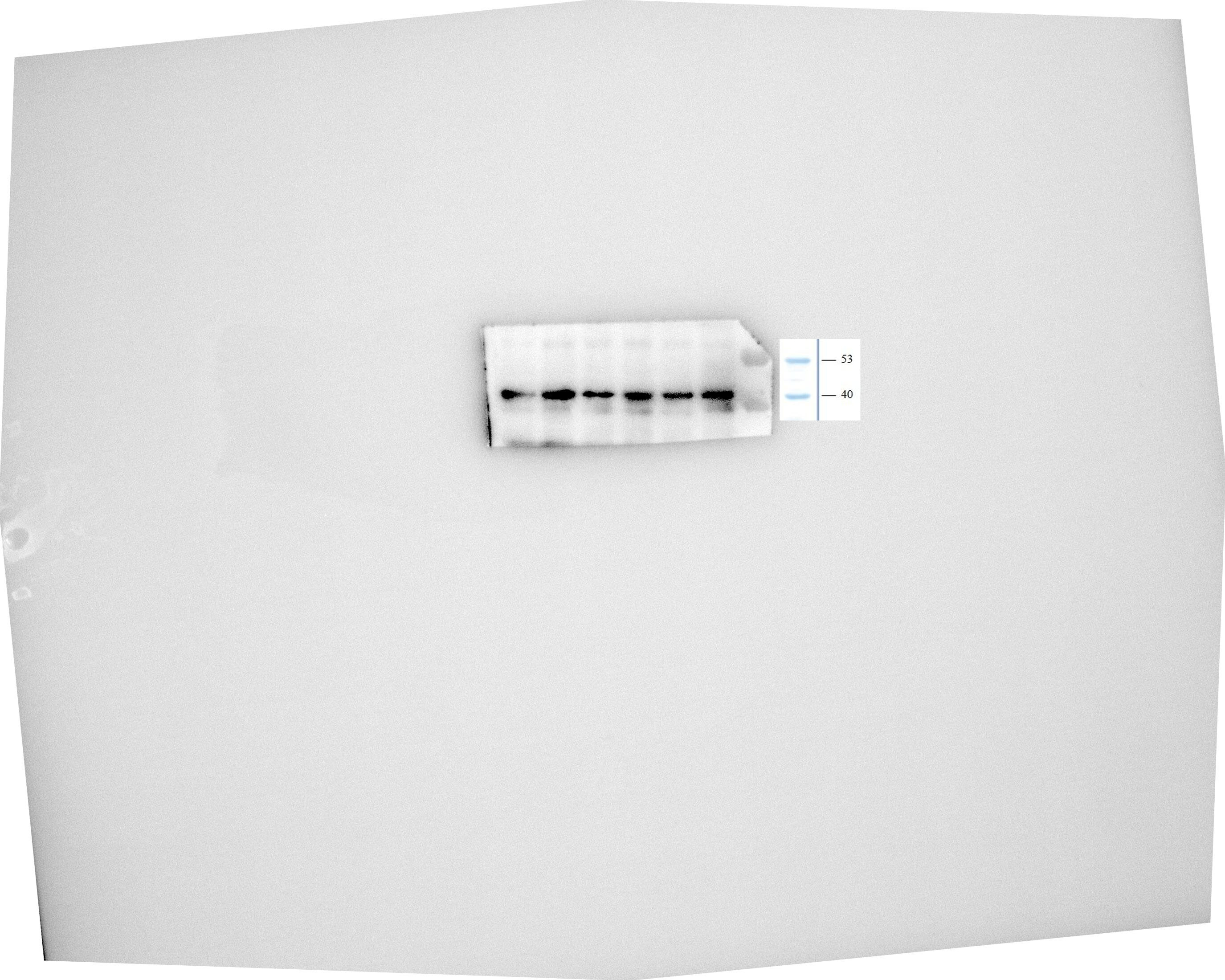

Supplement: Supplemental Information 5 [file peerj-11-15629-s005.zip › WB -Uncropped/MAPK12 ┐╜▒┤.jpg]

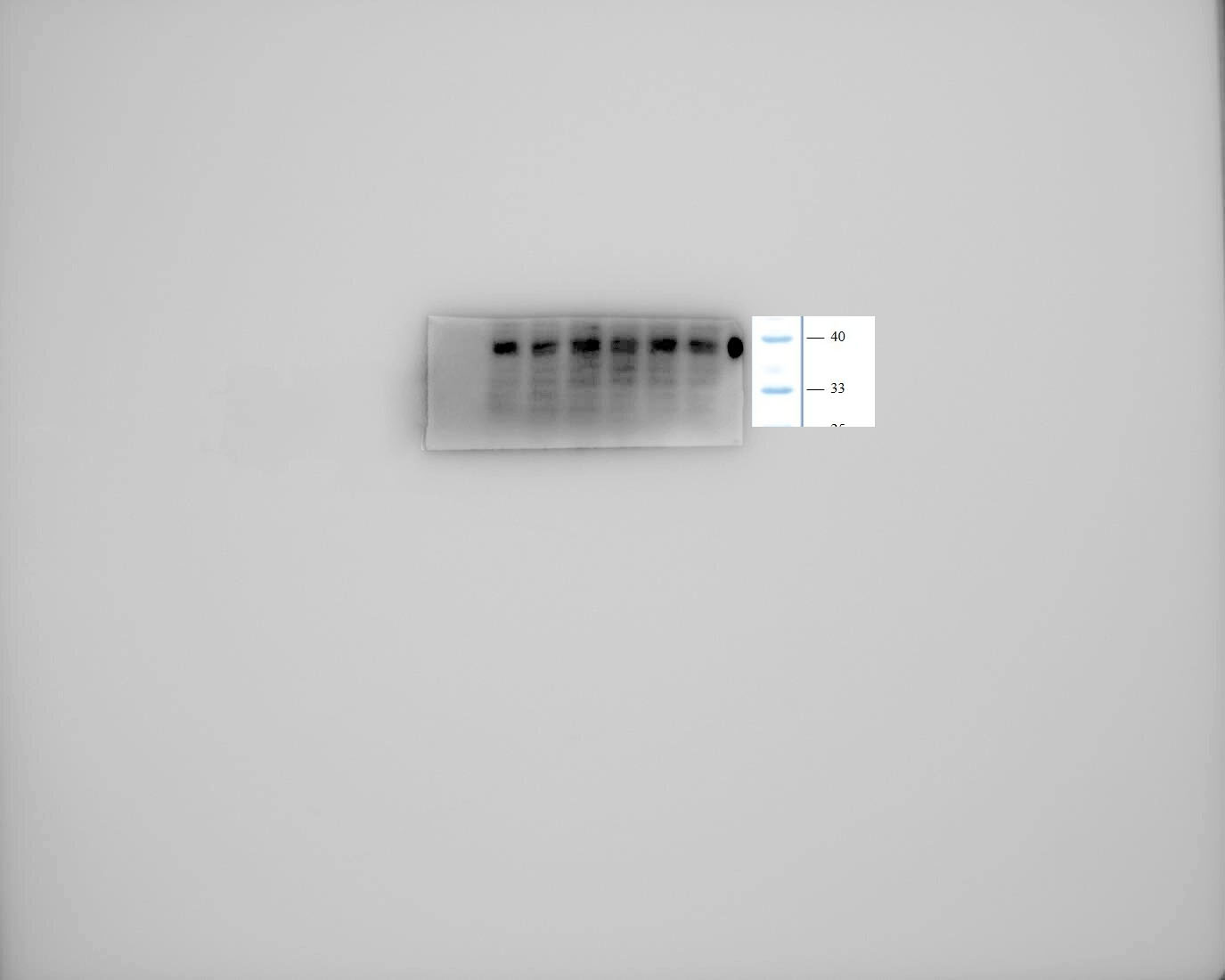

Supplement: Supplemental Information 5 [file peerj-11-15629-s005.zip › WB -Uncropped/MT-ND1 ┐╜▒┤.jpg]

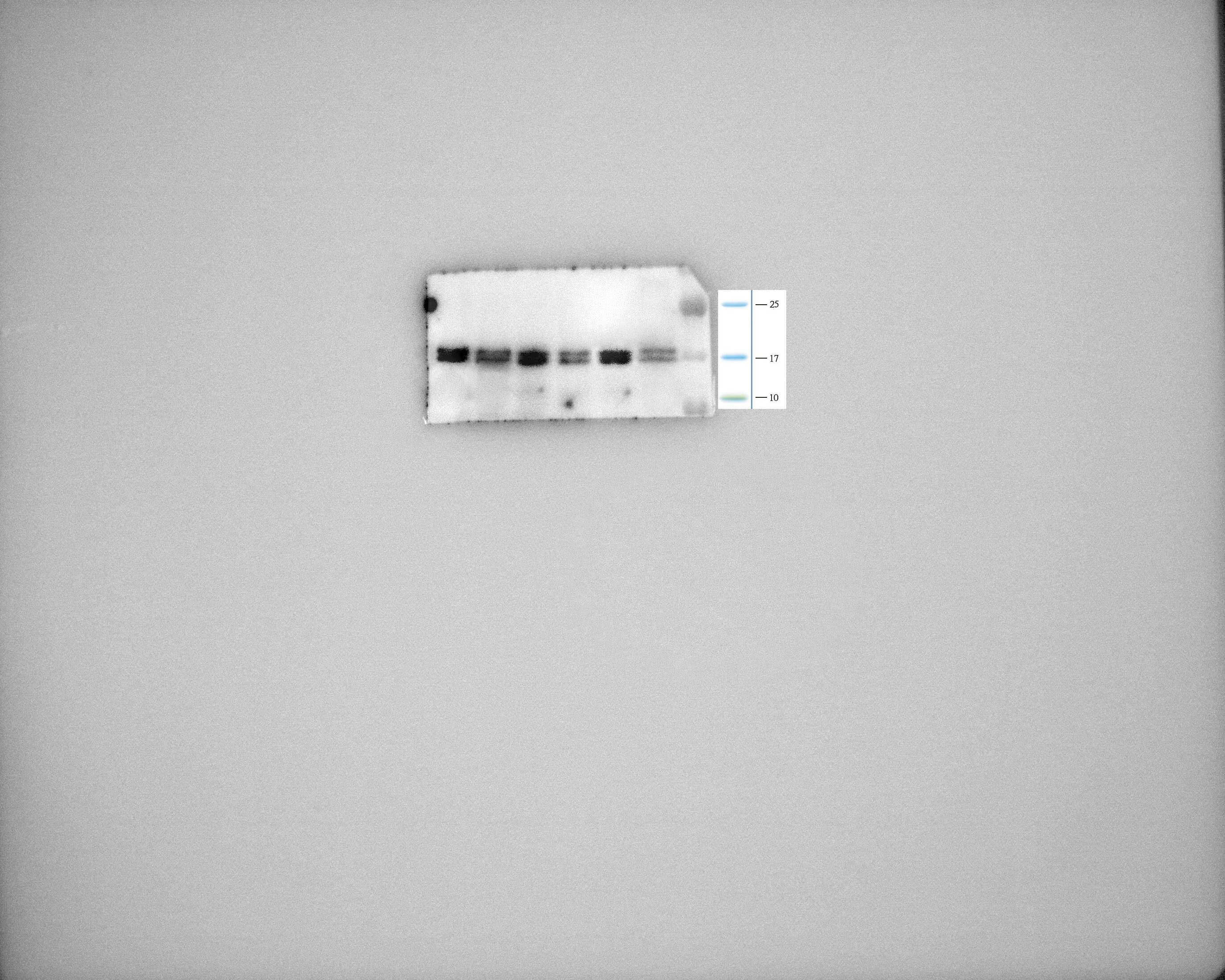

Supplement: Supplemental Information 5 [file peerj-11-15629-s005.zip › WB -Uncropped/UBA52 ┐╜▒┤.jpg]
